# Sonochemical Assisted Solvothermal Synthesis of Gallium Oxynitride Nanosheets and their Solar-Driven Photoelectrochemical Water-Splitting Applications

**DOI:** 10.1038/srep32319

**Published:** 2016-08-26

**Authors:** Naseer Iqbal, Ibrahim Khan, Zain H. Yamani, Ahsanullhaq Qurashi

**Affiliations:** 1 Center of Research Excellence in Nanotechnology, King Fahd University of Petroleum and Minerals, Dhahran, 31261, Saudi Arabia; 2 Department of Biosciences, COMSATS Institute of Information Technology, Park Road, Chak Shahzad, Islamabad, 45550, Pakistan; 3 Department of Chemistry, King Fahd University of Petroleum and Minerals, Dhahran, 31261, Saudi Arabia; 4 Department of Physics, King Fahd University of Petroleum and Minerals, Dhahran, 31261, Saudi Arabia

## Abstract

Gallium oxynitride (GaON) nanosheets for photoelectrochemical (PEC) analysis are synthesized via direct solvothermal approach. Their FE-SEM revealed nanosheets morphology of GaON prepared at a reaction time of 24 hours at 180 °C. The elemental composition and mapping of Ga, O and N are carried out through electron dispersive X-ray spectroscopy (EDX). The cubic structure of GaON nanosheets is elucidated by X-ray diffraction (XRD)analysis. The X-ray Photoelectron Spectroscopy (XPS) further confirms Ga, O and N in their respective ratios and states. The optical properties of GaON nanosheets are evaluated via UV-Visible, Photoluminescence (PL) and Raman spectroscopy’s. The band gap energy of ~1.9 eV is calculated from both absorption and diffused reflectance spectroscopy’s which showed stronger *p-d* repulsions in the Ga (3d) and N (2p) orbitals. This effect and chemical nitridation caused upward shift of valence band and band gap reduction. The GaON nanosheets are investigated for PEC studies in a standard three electrode system under 1 Sun irradiation in 0.5 M Na_2_SO_4_. The photocurrent generation, oxidation and reduction reactions during the measurements are observed by Chronoampereometry, linear sweep Voltametry (LSV) and Cyclic Voltametry (CV) respectively. Henceforward, these GaON nanosheets can be used as potential photocatalyts for solar water splitting.

Gallium compounds^1^,^2^,^3^,^4^,^5^ including gallium nitride (GaN), gallium oxide (Ga_2_O_3_) and more recently gallium oxynitrides (GaON/Ga_3_O_3_N/Ga_*x*_O_*y*_N_*z*_) have been known for their semiconducting and optoelectronic properties^6^,^7^,^8^,^9^. These gallium materials in different morphologies have enlightened ample of applications e.g. High electron mobility transistors (HEMTs)^10^, UV-Blue light emitting diode and laser diodes^11^, logic gates^12^,^13^, field effect transistors (FET)^14^ and gas sensing devices^15^. Despite of their diverse applications, very limited gallium oxynitride (GaON) compounds have been reported for solar water splitting studies^16^,^17^. Synthesis of GaON can be of significant interest to understand its intriguing properties. Formation of a direct band gap spinal structured gallium oxynitride (Ga_3_O_3_N) material^18^ has been reported within a gallium oxide and gallium nitride system with potential electronic properties^18^,^19^. Similarly, cubic gallium oxynitride crystal phase material of composition Ga_2.8_O_3.5_N_0.5_ is also carried out in a metastable state during the formation of GaN thin films by a chemical approach^20^,^21^.

Literature revealed that GaON can be prepared from both GaN and Ga_2_O_3_ by oxidation and nitridation/ammonolysis respectively. However, ammonolysis of Ga_2_O_3_ is favored as compared to the oxidation of GaN, because the subsequent method revokes the original structure via homogeneous formation of GaN^22^. Though, there are many factors that remained unclear in formation of GaON from ammonolysis route where a complete Ga_2_O_3_ layer growth over GaN crystal was observed^22^.Hu *et al*.^17^ presented a strategy in which they considered Ga(OH)_3_ as more appropriate precursor for GaON synthesisth an Ga_2_O_3_ and developed a visible-light active wurtzite-like gallium oxynitride (GaON) photocatalyst by nitridation of crystalline Ga(OH)_3_ with NH_3_ at elevated temperatures (550 and 900 °C). Their experimentation revealed the utilization of unoccupied 12-coordinate sites in crystal lattice of Ga(OH)_3_ that expedited ionic transportation during the nitridation. As incorporation of nitrogen is particularly helpful in reducing band gap of photocatalyst^16^ hence the GaON prepared showed band gap energies in the range of 2.2 eV to 2.8 eV. In addition, GaON showed superior PEC properties for production of H_2_ and O_2_ gases from methanol and silver nitrate (AgNO_3_) solutions respectively under visible light irradiation.

Since photocatalyts based solar water splitting for H_2_ and O_2_ production requires a suitable material with considerable band gap energy that could facilitate the electron mobility in an effective way^16^. Generally nitrides or oxynitride photocatalytic materials involving *d*^*0*^ or d^*10*^ electronic configuration with band gap energy below 3 eV are considered efficient for solar water splitting^23^,^24^. In case of *d*^*10*^ photocatalytic materials, the occurrence of conduction band of semiconductor is considered by hybridization of *sp* orbitals with large band dispersion. Thus observed high electron mobility and enhanced photoelectrochemical characteristics^17^,^25^. Group III nitrides such as GaN with band gap of 3.4 eV and wurtzite morphology showed limited photocatalytic water splitting. Its wide band gap can be tuned by doping metallic (M) nuclei (Zn, Ru) or preparing its oxynitride^17^,^26^. In such photocatalyts, the existence of M3d and N2p orbitals in the upper valence band provide *p-d* repulsions as a result upward switch of valence band ensued band gap reduction. Besides this the large dispersion of hybridized M3d, N2p, and O2p orbitals in doped nitrides or oxynitrides possibly augmented photogenerated hole mobility in valence band and thus further promoted photocatalytic activity^17^,^26^,^27^,^28^.

Herein, our research focuses on Gallium oxynitride (GaON),fabricated from gallium metal and organic diamine in a single-step at 180 °C and at different reaction times following sonochemical and hydrothermal approaches^7^,^29^. The surface morphology under FE-SEM showed formation of uniform nanosheets containing nanopores and defects in the structure obtained at a reaction time of 24 hours. The as prepared GaON nanosheets at 180 °C for 24 hours is characterized by EDX, XRD and XPS for elemental and structural insights. Furthermore, its optical properties are examined by FTIR, Raman UV-Visible spectroscopy’s and Photoluminescence studies. In addition to aforementioned characterization of as prepared GaON nanosheets at 180 °C for 24 hours, we carried out FE-SEM, XRD studies of GaON prepared at 3, 6 and 12 hours reaction time. However, based on our experimental observations we conducted photoelectrochemical studies of GaON prepared at 180 °C for 12 hours, 24 hours as well as GaON prepared at 180 °C for 24 hours and annealed at 500 °C respectively. Thin films of GaON samples are deposited over FTO substrates and tested by employing standard three electrode system for photoelectrochemical studies under chopped solar (1 Sun) irradiation source.

## Results and Discussions

**Figure 1:**
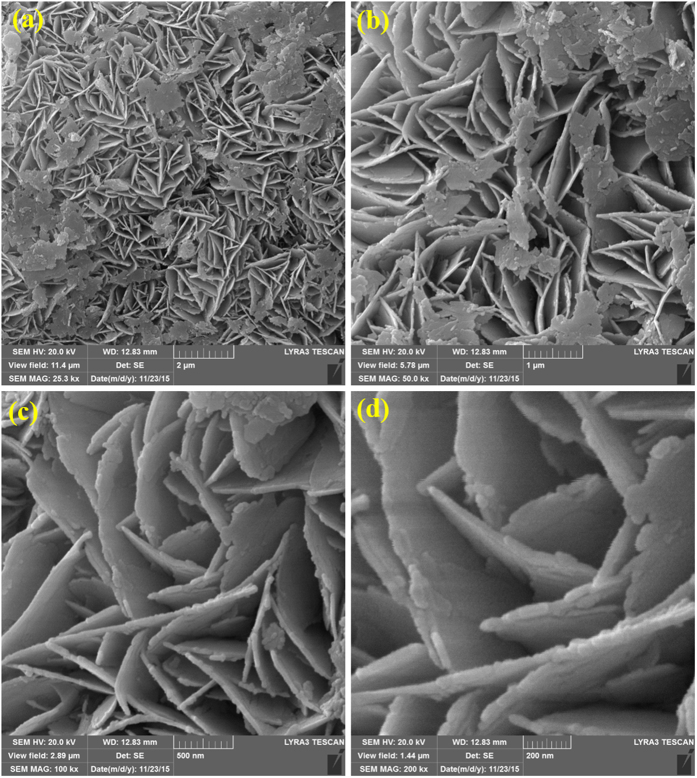
SEM images of GaON nanosheets (**a,b**) low resolution micrographs at 2μm and 1μm showing growth patterns of nanosheets (**c,d**) higher magnification micrographs show internal texture and defects attributing towards availability of high surface area.

**Figure 2:**
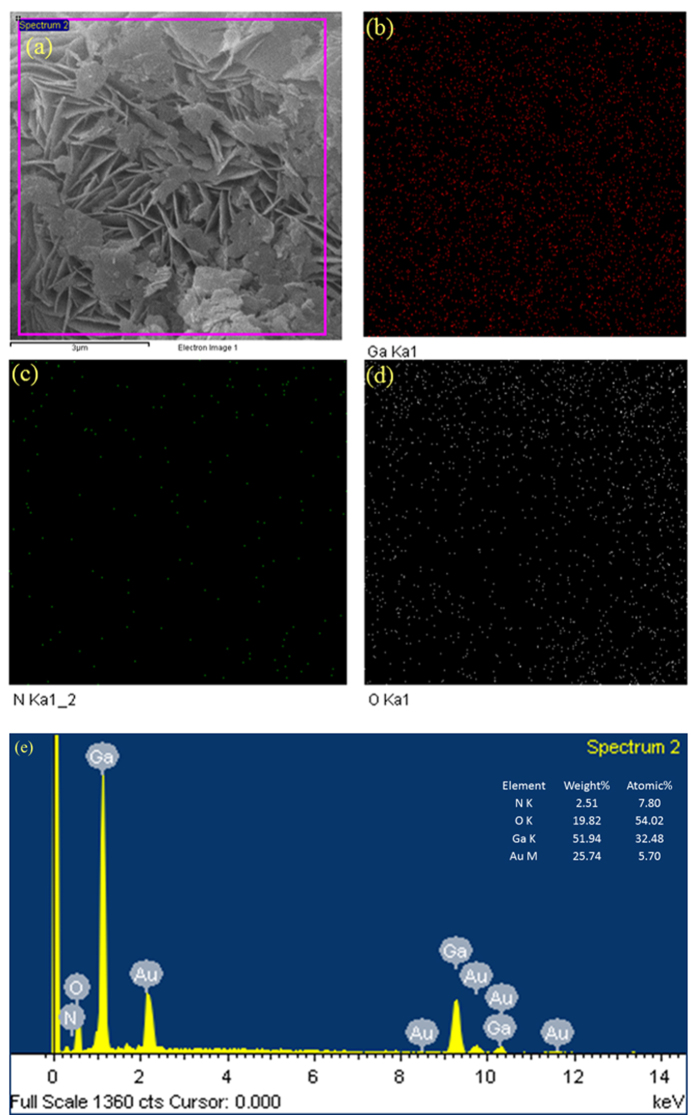
EDX elemental composition and mapping of the GaON nanosheets. Where (**a**) showing Fe-SEM micrograph undertaken for elemental composition analysis (**b–d**) presenting elemental maps of Ga, O and N respectively. (**e**) Expressing the spectrum investigated for atomic composition, the onset in (**e**) also shows their corresponding weight and atomic percentages.

The FE-SEM analysis at first explored nanosheets like morphology of our proposed GaON material through a solvothermal reaction at slightly better temperature of 180 °C for 24 hours. This strategy is straightforward and facile in comparison to high temperature synthesis that are reported elsewhere^19^,^22^,^26^,^30^. To our best of knowledge, this kind of morphology with one-step synthetic strategy and a single GaON phase photocatalyts for solar water splitting is not reported. We carried out FE-SEM studies as shown in Fig. 1, the FE-SEM micrographs presented a uniform growth of variable size and width of GaON nanosheets. Moreover, we performed FE-SEM analysis for as prepared GaON at different reaction times (3, 6 & 12 hours @ 180 °C) as well as for GaON prepared at 24 hours and annealed at 500 °C for 4 hours, the data is presented in Fig. S1 of Supplementary information. The Fig. 1(a,b), shows lower magnification micrographs of GaON nanosheets, a clear arrangement of nanosheets of variable size is explicable. Whereas Fig. 1(c,d) further elucidate high resolution micrographs of GaON nanosheets. It is evident from Fig. 1(d) that the thickness of the nanosheets lies between 15–30 nm whilst the length is variable we could assume between 400–1000 nm. Furthermore, the petals like flower arrangement of nanosheets has made its morphology inimitable with interfacial distance of 100–500 nm. Moreover, the nanosheets surface also showed nanoporesas well as ordered defects that are supposed to show some shifts in XRD patterns in addition to better photoelectrochemical properties. This morphology is attributed to the *in-situ* incorporation of N and O in the chemical reaction of Ga-ethylenediamine-water complex through a hydrothermal reaction at 180 °C. In order to confirm the elemental existence of each component and our assumed product, energy dispersive spectroscopy (EDX) is performed. The Fig. 2 shows elemental mapping/distribution encompassing composition of each Gallium, Oxygen and Nitrogen entities in the nanosheets and EDX spectrum respectively. Their weigh and atomic ratios are well justifiable for the single phase as prepared GaON nanosheets, however the appearance of gold in the Fig. 2(e) spectrum is because of gold coating used for FE-SEM and EDX analysis.

**Figure 3:**
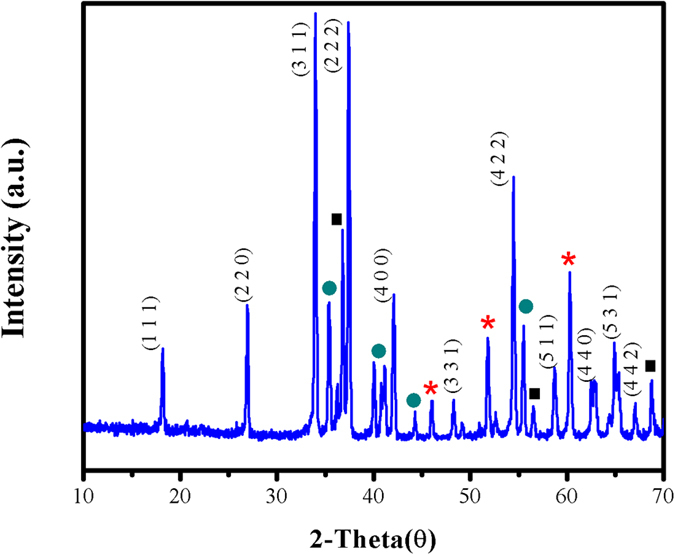
XRD patterns of as prepared GaON nanosheets prepared via a solvothermal route @ 180 °C for 24 hours. Where (_*_), (■), (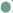) represents new XRD peaks, patterns of XRD compared with GaN and Ga_2_O_3_ respectively.

The powder XRD analysis enlightened diffraction patterns and crystalline phase of as prepared GaON nanosheets at 180 °C for 24 hours. Figure 3 shows, characteristic peaks that could be indexed partially and assigned a cubic crystal structure with space group Fd-3m(227) and composition of Ga_*2.79*_O_*3.05*_N_*0.76*_ via JSPDF card number 01-078-8634 from XRD data base and literature^31^. Furthermore, the XRD patterns of GaON prepared at 180 °C for 3, 6 and 12 hours as well as GaON nanosheets prepared at 180 °C for 24 hours and annealed at 500 °C for 4 hours are compared in Fig. S2 of Supplementary information. In Fig. 3, the insights to XRD spectrum exposed highest intensity peaks corresponding to cubic GaON nanosheets in their most probable forms which are recorded at 2 theta (θ) range of 27°, 34°, 37°, 42° and 54° with indices 220, 311, 222, 400 and 422 respectively. However the median to lower range XRD patterns observed at 2 theta (θ) values i.e., 17°,48°, 58°, 63°,65° and 67° with indices 111, 331, 511, 440, 531 and 442 also substantiate with the cubic crystal structure XRD patterns of Ga_*2.79*_O_*3.05*_N_*0.76*_ via JSPDF card number 01-078-8634. The peak shifting and appearance of new patterns(red star pointed in Fig. 3) observed are assumed due to formation of single phase GaON. In addition, some peaks in XRD spectra (tagged with green circle and black square) are also compared with GaN and Ga_2_O_3_ XRD patterns in the data base. However their peaks showed low intensities and slight shifting in the XRD region therefore no crystallization of the two stable phases GaN and Ga_2_O_3_ could be observed. These differences in the lattice parameters are obvious depending upon the experimental conditions commenced since cubic gallium oxynitride has been known to exist in a metastable state during oxidation or ammonolysis of GaN and Ga_2_O_3_ respectively^6^,^7^,^19^,^22^,^30^,^32^. Consequently, all such broad reflections in XRD are indicative of crystallinity as well as somewhat ordered defects in composition of as prepared GaON nanosheetswhich are also depicted in FE-SEM and EDX studies.

**Figure 4:**
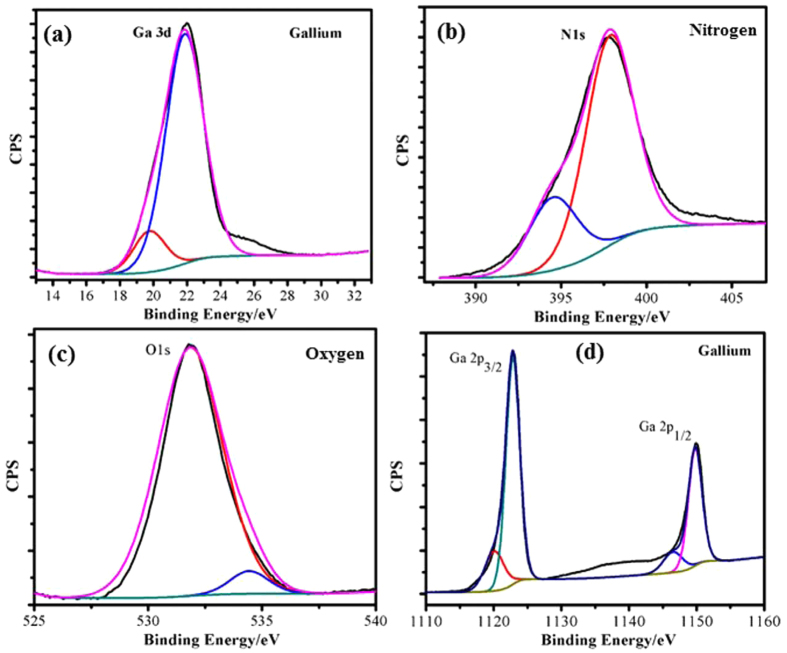
XPS photoelectron spectroscopy of as synthesized GaON nanosheets at 180 °C @ 24 hours presenting binding energy profiles for (**a**) Ga 3d (**b**) N1s (**c**) O1s and (**d**) Ga2p_3/2_ & Ga 2p_1/2_.

To further elucidate the structure of GaON nanosheets, XPS measurements are carried out, all the peaks observed are fitted according to the standard methods^17^. Figure 4(a–d) show XPS binding energy profiles of various chemical states of Ga, O and N. A deeper insight to Fig. 4(a) gave clue about two peaks for Ga 3 d at binding energies 19.77 eV and 21.87 eV respectively that also validated with the literature values for Ga compounds^17^,^33^,^34^,^35^. The shifting of these peaks to higher values as well as appearance of d-p repulsions is obviously due to incorporation of nitrogen/oxygen in the gallium metallic center from the reaction precursors. Hu *et al*. reported this in their studies^17^, furthermore, they explained strong hybridization between the valence orbitals of Ga, N and O atoms that can be observed in binding energy range of 0–14 eV and assigned to hybrid Ga4p-N2p, Ga4s-N2p, and Ga4s-O2p chemical states^36^,^37^,^38^. These hybrid states are also observed in our as prepared GaON nanosheets at 24 hours and are presented in Fig. S3 along with XPS survey spectra in Supplementary information. The occurrence of this phenomenon as evident from XPS spectra also strengthen the efficacy of our straightforward synthetic approach that incorporated nitrogen and oxygen simultaneously during reaction between Gallium-ethylenediamine-water at reaction temperature of 180 °C.

Figure 4(b) provides extended information of fitted peaks recorded at binding energy range 397.86 eV (main peak) and 394.52 eV (shoulder peak) which are assigned to Nitrogen (1s). This assignment is straight forward for existence of Ga-N bond as reported for various nitrogen containing Ga materials^39^,^40^,^41^,^42^. Similarly, the Fig. 4(c) shows high resolution spectra of fitted peaks near binding energies 531.86–534.44 eV assigned for core level O1s^43^,^44^,^45^,^46^,^47^. The intense peak for O1s is observed at 531.86 eV whereas the shoulder peak appears at 534.44 eV.Literature^48^,^49^ revealed that the main O1s peak in Gallium oxide occurs at a binding energy of ~531 eV and supports the formation of Ga-O bonding with the highest oxidation state of Ga is (Ga^3+^). Figure 4(d) shows the doublet peaks observed at binding energies of 1120.11–1122.84 eV and 1146.51–1149.79 eV that are characteristics for Gallium (Ga) and thus assigned as Ga 2P_3/2_ and Ga 2P_1/2_ respectively^50^. These binding energies are different from Ga 2p levels in metallic Gallium i.e., 1117.0 and 1144.0 eV for Ga 2p_3/2_ and Ga 2p_1/2_, respectively^51^. Nonetheless, this reflects a positive shift in our as prepared GaON material which is attributed to redistribution of electronic charge around the reacting atoms^52^,^53^. Therefore, the difference in Ga chemical bonding causes a binding energy shift that can be used to extract chemical states information. This further enlighten oxidation state of Ga metal in the growing GaON nanosheets in its highest valence state (Ga^3+^)^50^,^54^. The XPS studies thus comply the formation of GaON nanosheets via our straightforward and facile approach at 180 °C for 24 hours that can be used as photoactive material for enhanced water splitting performance.

**Figure 5:**
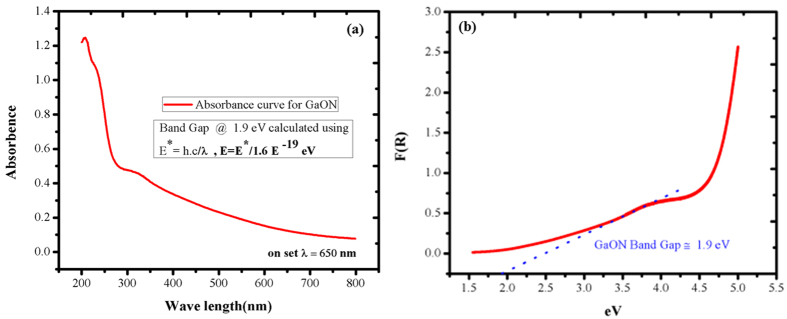
UV-Vis spectroscopic studies of as prepared GaON nanosheets at 180 °C @ 24 hours in powder form (**a**) UV/Vis absorption curve (**b**) diffused reflectance spectrum for band gap energy calculation.

The absorbance and diffused reflectance studies are performed by UV-VIS spectroscopy in order to estimate band gap energy of as prepared GaON nanosheets synthesized from a solvothermal route. The powder sample is used for absorbance and diffused reflectance analysis. Figure 5(a) shows the absorbance curve, the spectrum shows a strong cut-off wave length observed at 650 nm where comparatively low absorbance is observed. This wavelength is used to calculate the band gap energy of as prepared GaON nanosheets according to the methods reported elsewhere^55^,^56^. The band energy is calculated around ~1.9 eV, further to confirm this band gap energy we evaluated the diffused reflectance spectroscopy as shown in Fig. 5(b) for band gap calculation. Thus the band gap estimated from this also comparable with that observed from absorbance curve. The reduction of band gap energy is attributed to incorporation of nitrogen and the existence of Ga*3d* and N*2p* orbitals in the upper valence band of GaON that encouraged more *p-d* repulsions hence caused an upward shift in its valence band^16^,^17^,^26^.

**Figure 6:**
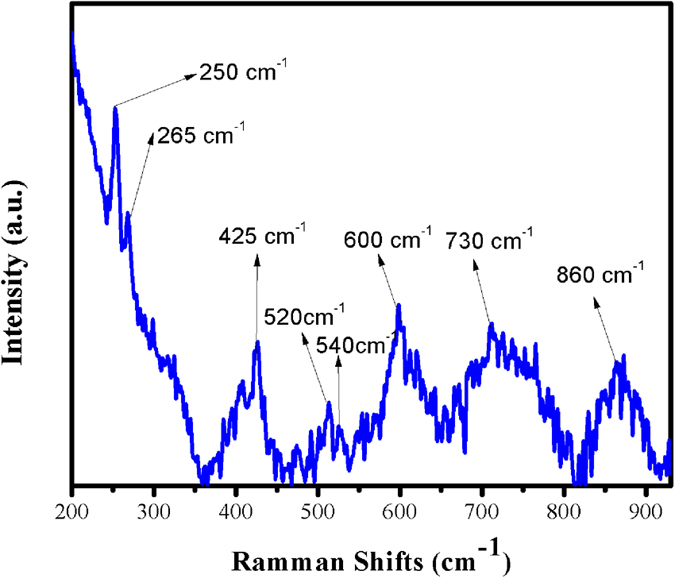
Raman spectroscopy of as prepared single phase GaON nanosheets at 180 °C @ 24 hours through a solvothermal approach.

In order to elucidate the structural composition of the cubic GaON nanosheets we investigated it for Raman, FTIR and Photo luminescence studies (FT-IR and PL are discussed in Supplementary information Fig. S4 and Fig. S5 respectively). The Raman spectra of GaON is shown in Fig. 6. The gallium oxynitride cubic phase showed broad Raman shifts that symbolizes significant disorder among O and N atoms in the cubic structure and/or the presence of cation (Ga^3+^) or anion vacancies^7^ during the solvothermal synthesis of GaON. The maxima of these good intensity broad bands are observed around 250 cm^−1^, 425 cm^−1^, 600 cm^−1^ 730 cm^−1^and 860 cm^−1^. However, some narrow bands of medium to lower intensity are also observed at 265 cm^−1^, 520 cm^−1^and 540 cm^−1^ respectively. These fingerprints phonon frequencies are comparatively in good agreement with those reported in literature for Ga_3_O_3_N^6^,^7^. Soignard *et al*.^6^ carried out theoretical calculations by first principles local density approximation (LDA) methods for a Ga_3_O_3_N pseudocubic R3hm model structure. They proposed nine Raman-active modes (4A_*1g*_ + 5E_*g*_), with zone-center frequencies calculated at 213, 219, 367, 379, 499, 512, 634, 647, and 782 cm^−1^ respectively. However they concluded that the zone-center modes acted as poles for broadened spectra with complete vibrational density of states (*v*DOS). Hence, their expected mode frequencies for pseudocubic Ga_3_O_3_N were grouped into five phonon frequencies i.e., 216, 373, 506, 640, and 782 cm^−1^. Thus for an ideal spinal structure gallium oxynitride one should expect these phonon frequencies with slight acceptable variations depending upon the nature of chemical reaction undertaken and morphology achieved. This perception is further supported by Oberlaender *et al*.^7^ for their cubic spinal-type Ga_3_O_3_N material. They described five active Raman modes i.e., *Г*_Raman_ = 3T_*2g*_ + 1E_*g*_ + 1 A_*1g*_ and assigned their phonon frequencies for the respective Raman bands. The broadness of Raman bands is attributed to the high disorder of nitrogen and oxygen atoms in the anionic cites during growth of GaON as described earlier i.e., above 8% of nitrogen in our as synthesized GaON. In light of these experimental and theoretical studies the high intensity Raman bands in our as synthesized GaON nanosheets are assigned in the following order: Phonon frequency/modes, 250 cm^−1^(T_*2g*_), 425 cm^−1^(E_*g*_), 600 cm^−1^ (T_*2g*_), 730 cm^−1^ (T_*2g*_) and, 860 cm^−1^ (A_*1g*_).

**Figure 7:**
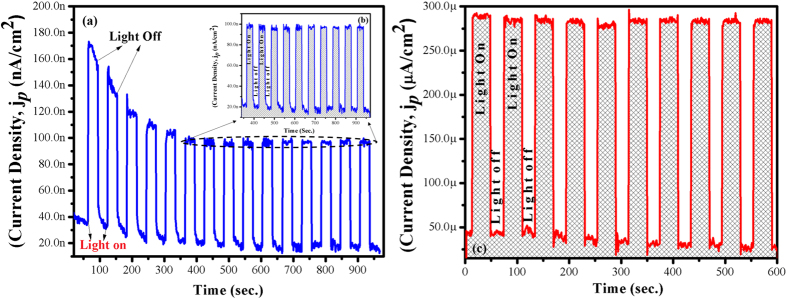
Chronoampereometry showing photocurrent densities for as prepared GaON nanosheets @ 180 °C for 24 hours. (**a**) Photocurrent recorded at 0 V applied potential (**b**) onset shows photocurrent stability for longer period of time and (**c**) photoresponse recorded at −1.2 V applied potential. All measurements are observed under chopped on/off light from a solar simulator of AM 1.5G filter and 100 mW cm^−2^(1Sun) light intensity of Xe lamp.

**Figure 8:**
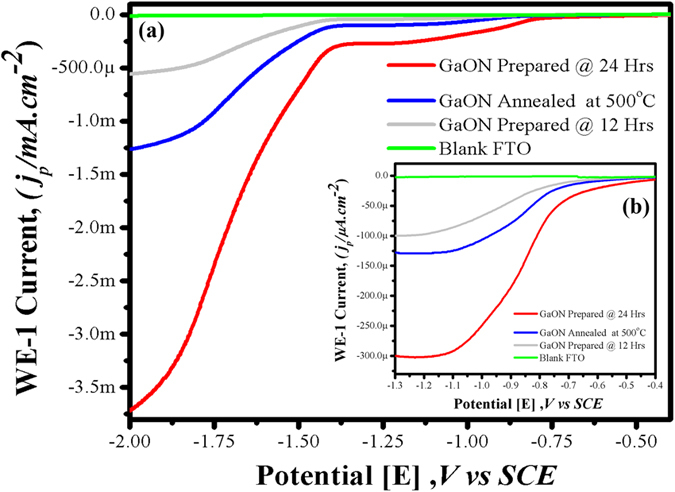
Linear sweep voltammograms of GaON samples coated on FTO glass under simulated solar light (1 Sun) with scan rate of 100 mV/sec in a standard three electrode system, SCE, Pt-cathode and GaON coated FTOs as anode, (**a**) shows comparative photocurrent densities of GaON prepared at different reaction conditions, photocurrent density in mA range is recorded against applied potential (−0.4 to −2.0) whereas the onset (**b**) is presenting μA range photocurrent density, when the applied potential is in the range of −0.4 to −1.3 V respectively.

The FTO coated GaON nanosheets are used for probing photoelectrochemical performance in a standard three electrode setup i.e., a reference standard calomel electrode (SCE), platinum counter electrode and GaON coated FTO photoanode as working electrode respectively. The PEC cell containing FTO/GaON photoanode and other respective electrodes are dipped in 0.5 M Na_2_SO_4_ electrolyte solution at neutral pH (7) and are exposed to illumination source (1 Sun) in an on/off fashion with regular intervals of time to investigate the photoresponse of the GaON nanosheets. Figure 7 (a) shows the J*p* – t profile where photocurrent generated is plotted as function of time. The current produced from this device is in the range of nanoamperes (140 nA–80 nA at OCP ~ 0 V = applied potential). Whereas this photocurrent density substantially increased to ~240 μA at applied potential of −1.2 V as shown in Fig. 7(c). Besides this, we observed enhanced photocurrent density in mA range i.e., ~3.75 mA at higher potential values recorded from GaON nanosheets in linear sweep voltametry (LSV) experiments as presented in Fig. 8. In chronoampereometry, the first *J*_p_ (photocurrent density) spike is recorded to a higher value upon GaON exposure to 1 Sun irradiation but later on relaxed to a stable plateau that explicit the significant stability of the GaON film with the passage of time as can be seen in Fig. 7(a), in onset Fig. 7(b,c) respectively. The initial decline in the photocurrent response is observed in course of instituting equilibrium between FTO coated electrode layer and the electrolyte solution upon instant exposure to solar irradiation source. Furthermore, these chronoampereometric measurements carried out at different applied potentials (0 V and −1.2 V) for longer time span showed significant stability for several minutes, as also presented in Supplementary information Fig. S6. Inprogression of PEC measurements, shifting of the *J*_p_ − *t* profile to its normal baseline under dark (no illumination) exhibited a reversible responsehence acclaimed that photocurrent generation is solely due to solar driven water splitting reaction. Nonetheless, the structural morphology, reduced band gap and a straight forward synthetic approach are advantageous features of as prepared GaON nanosheets that corroborate to better PEC characteristics.

**Figure 9:**
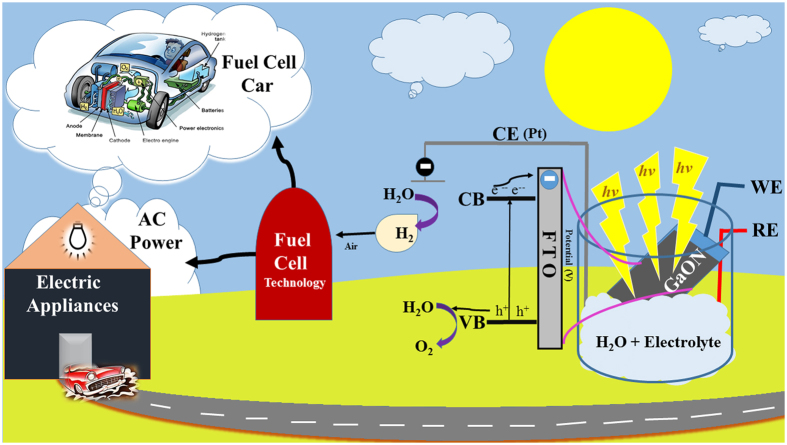
A schematic illustration of photo-electrochemical solar-driven hydrogen production device via water splitting.

The Fig. 8 shows linear sweep voltammetry (LSV) curves of the as prepared GaON at 180 °C for 12 hours, 24 hours and GaON annealed sample in a standard three electrode configuration using 0.5 M Na_2_SO_4_ as electrolyte. Figure 8(a) shows comparative LSV profiles of bare FTO and FTO coated with different GaON samples tested at applied potential range versus SCE under 1 Sun simulated solar light. The as prepared GaON nanosheets at 180 °C for 24 hours sample showed highest photocurrent of magnitude 3.75 mA whereas its annealed GaON nanosheets produced more than 2 fold lower photocurrent i.e., 1.25 mA. Likewise, GaON sample obtained at 180 °C for 12 hours generated photocurrent in ~500 μA range under the applied potential, this current density is more than three times lower as compared to current density observed from as prepared GaON nanosheets at 180 °C for 24 hours. The most probable reasons of this decreased photocurrent recorded from GaON obtained at 12 hours and GaON annealed samples are attributed to in complete nanosheets growth and deformation of nanosheets morphology which resulted in thickening and distortion of the nanoflakes hence caused reduced electron mobility and charge separation respectively. An additional LSV measurement is presented in Fig. 8(b) onset. The as prepared GaON nanosheets at 180 °C for 24 hours showed highest photocurrent in comparison to competing GaON samples when the applied potential is in the range of −0.4 to −1.3 V. While further insights show that a photocurrent of ~250 μA is observed at applied potential −1.2 V which elucidate solar water splitting and H_2_ production by GaON under the same experimental conditions as discussed in chronoampereometric studies earlier and presented in Fig. 7(c). In contrast the LSV curves obtained for GaON 12 hours and GaON annealed samples showed significantly decreased photoresponse. To further support our preceding findings for as prepared GaON nanosheets at 180 °C for 24 hours sample we also carried out its cyclic voltammetry (CV) (details provided in Supplementary information Fig. S7). The reduction potential observed in CV curves is between −1.1 V to −1.2 V and photocurrent of ~280 μA is also recorded. This indicated that reduction reaction i.e., hydrogen generation is favorable by Gallium oxynitride under solar driven photocatalytic water splitting. Thus in all PEC experiments, the enhanced photocurrent generation from as prepared GaON nanosheets at 180 °C for 24 hours sample is assumed due to nanoporous texture and appearance of defects on the surfaces of GaON nanosheets. Furthermore, the *in-situ* embedding of nitrogen and oxygen to Gallium metal during its solvothermal synthesis not only causes defects on Gallium sites but also leave substantial O/N disorder. This in turn helps in better electron mobility and minimal electron hole recombination.The schematic for electron hole pair generation, charge separation mechanisms and hydrogen production under solar light with perspective future green energy fuel are shown in Fig. 9.

In summary, the as prepared GaON nanosheets by sonochemically assisted solvothermal process at comparatively low temperature showed significantly enhanced photoelectrochemical properties. The XRD, XPS and Raman spectroscopy’s revealed the cubic crystal structure in which gallium exists in it highest chemical state of Ga (3^+^) and showed further interaction with N and O in a very disordered way thus cause nanopores and defects on the surface. This ultimately helped in better optical and photoelectrochemical properties by providing better electron mobility and minimal electron hole recombination. The facile synthetic route and the nanosheets like morphology are the advantageous features of GaON which also contributed in enhanced physicochemical properties. Furthermore, the appreciable photoelectrochemical characteristics such as, generation of photocurrent, reversible response, stability over certain period of time etc. are the key parameters of this single phase GaON nanosheets material that make it significantly better in the series of gallium compounds used for solar water splitting. Nevertheless, the photoelectrochemical properties of such GaON materials can be further enhanced by tuning its material’s chemistry such as by doping different other metals with GaON via physical methods or incorporation of certain co-catalyst in GaON nanosheets by chemical process or by adopting an electrochemical method to develop its well-aligned hetero nanostructures.

## Methods

### Materials Synthesis

All the chemicals and reagents i.e., Gallium metal, ethylene diamine, sodium sulphate (Na_2_SO_4_), ethanol and acetone were purchased from Sigma Aldrich and used as received unless otherwise stated. For synthesis, few grams of Gallium metal are mixed with ethylenediamine and placed in an ultrasonic bath for an hour at 75 °C. During the ultrasonification, deionized water is added in 5 ml portions after every 5 min. time span. The appearance of black suspension showed the formation of Ga- ethylenediamine complex. After complete dissolution of gallium metal in ethylenediamine water solution, the reaction mixture is shifted into a stainless steel autoclave containing a Teflon vessel. The solvothermal reaction is carried out for 3, 6, 12 and 24 hours at 180 °C respectively. Afterwards the reaction mixture containing precipitates of Galliumoxynitride (GaON) in each case is centrifuged at 4000 RPM for 5 mins washed with ethanol and acetone respectively before drying in a vacuum oven at 100 °C for two hours. Furthermore, the as prepared GaON nanosheets at 180 °C for 24 hours is further annealed at 500 °C for 4 hours owing to its best morphology achieved for comparative PEC studies.

### Fabrication of FTO/GaON nanosheets based device and photoelectrochemical setup

Photoelectrochemical characteristics of the GaON nanosheets are investigated using FTO conducting glass substrates. Initially, FTO glass substrates are washed by acetone (10 min) and water (10 min) respectively with continuous ultrasonification. In the next step slurry of GaON nanosheets samples (taken from GaON obtained at 12 hours, 24 hours reaction times and 24 hours but annealed at 500 °C respectively) with ethanol are prepared and drop coated over the pre-treated FTO glass substrates to produce a smooth film. The FTO/GaON substrates are heated at 100 °C for 2 hours in order to evaporate the solvent and harden the GaON nanosheets layers over to FTO substrate that could withstand during the PEC measurements. The photoelectrochemical measurements are carried out by a three electrode system in 0.5 M Na_2_ SO_4_ (pH = 7) solution, where a Pt wire served as auxiliary electrode, FTO coated with different GaON substrate served as photoanode or working electrode and the standard calomel Ag/AgCl reference electrode (SCE). All the photoelectrochemical experiments are performed through Metrohm Autolab Potentiostat (PGSTAT302N) instrument. However, for Solar light in lab we used Oriel sol 3A class AAA solar simulator-Newport with following specifications i.e., power 100 mW.cm^−2^(1 Sun), IEC/JIS/ASTM certified containing 450 Watt Xenon lamp, Air Mass 1.5G Filter, UV cut off filter and 2 × 2 inch aperture for output beam. The data obtained from photoelectrochemical measurements is discussed according to the standard calomel electrode (SCE).

### Characterization of Gallium Oxynitride Nanostructures

The purity and crystalline phases of as synthesized GaON nanomaterials are characterized by X-ray diffraction (XRD) technique using a Benchtop MiniFlex -X-ray Diffraction instrument (Mini-XRD) from Rigaku (40 kV and 15 mA) in the range of 10–70° (2θ) at a scanning rate of 3° min-1 with CuK alpha radiation and wave length of 1.54060. The structural composition and elemental states involved in GaON formation are also verified by x-ray photoelectron spectroscopy (XPS) using PHI 5000 VersaProbe II spectrometer (UlVAC-PHI), employing Al Kα as the incident radiation source. The C1s (E = 284.5 eV) level was served as the internal standard. The morphology of the GaON nanomaterial is observed under TESCAN Lyra 3 Field Emission Dual Beam (Electron/Focused Ion Beam) system combined high‐end field‐emission scanning electron microscope (FE-SEM) also facilitated with EDX for elemental determination/mapping. Optical properties like absorption and diffused reflectance spectroscopy of GaON nanosheets are carried out using Agilent Cary 5000 high performance UV-Vis-NIR spectrophotometer containing praying mantis accessory with alignment tools and powder cell sample cups. *In situ* Raman spectroscopy of GaON is performed with laser (300 mW Green 532 nm) by iHR320 Horiba imaging spectrometer. The vibrational modes in the GaON are recorded by FT-IR 6700 Nicolet™ FT-IR spectrometer from Thermo Electron Corporation. The material is also tested for photoluminescence studies carried out with fluorolog-3 Imaging Spectrophotometer at an excitation wavelength of 350 nm and slit width of 2 nm.

## Additional Information

**How to cite this article**: Iqbal, N. *et al*. Sonochemical Assisted Solvothermal Synthesis of Gallium Oxynitride Nanosheets and their Solar-Driven Photoelectrochemical Water-Splitting Applications. *Sci. Rep.* **6**, 32319; doi: 10.1038/srep32319 (2016).

## Supplementary Material

Supplementary Information
