# Supplementary material for: Sonochemical Assisted Solvothermal Synthesis of Gallium Oxynitride Nanosheets and their Solar-Driven Photoelectrochemical Water-Splitting Applications
Source: Sci Rep. 2016 Aug 26;6:32319. doi: 10.1038/srep32319 (PMC5000014; doi:10.1038/srep32319)
Supplement: Supplementary Information [file srep32319-s1.pdf]

Supplementary Information:

Sonochemical Assisted Solvothermal Synthesis of Gallium Oxynitride Nanosheets and their Solar-Driven Photoelectrochemical Water-Splitting Applications

Naseer Iqbal[1, 2], Ibrahim Khan[1, 3], Zain H.Yamani[1, 4] and Ahsanullhaq Qurashi[1, 3*]

[1]*Center of Research Excellence in Nanotechnology, King Fahd University of Petroleum and Minerals, Dhahran, 31261, Saudi Arabia.*

[2]*Department of Biosciences, COMSATS Institute of Information Technology, Park Road, ChakShahzad, Islamabad, 45550, Pakistan.*

[3]*Department of Chemistry,King Fahd University of Petroleum andMinerals, Dhahran, 31261, Saudi Arabia.*

[4]*Department of Physics,King Fahd University of Petroleum andMinerals, Dhahran, 31261, Saudi Arabia.*

[*]*Corresponding author. Tel.: +966 (0)138607063. Email: ahsanulhaq@kfupm.edu.sa.*

1. FE-SEM Microscopy XRD Analysis of GAON prepared at different reaction times.

The FE-SEM microscopy ofGaON prepared at different reaction times is carried out in order to optimize the reaction time and to obtain better morphologyin association to high temperature synthesis reported in literature[1-4]. We observed that among all the samples, GaON prepared at 180 °Cfor24 hours showed uniform nanosheets like morphology under FE-SEM as compared to GaON prepared at 3, 6 and 12 hours respectively.

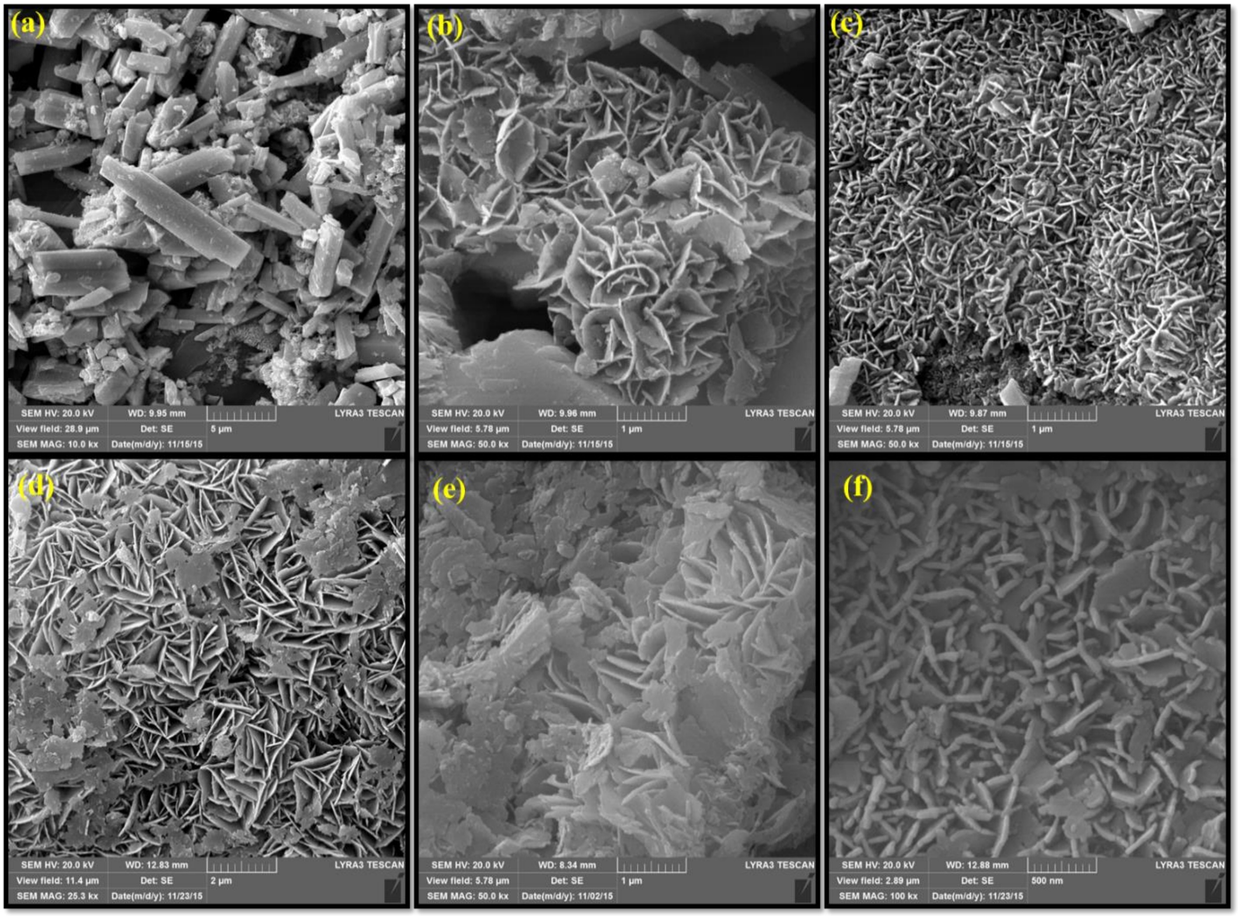

Figure S1: FE-SEM micrographs of GaON prepared at different reaction times as well as annealed sample. (a) Low resolution micrograph at reaction time 3 hours (b) at 6 hours the FE-SEM micrograph showed undeveloped GaON nanoflakes. (c) FE-SEM of GaON taken for reaction time 12 hours. (d) Low resolution micrograph showing uniform GaON nanoflakes

formation at 24 hours of reaction time (e) Deformation of GaON nanoflakes morphology of as prepared GaON at 24 hours and annealed at 500 °C for 4 hours (f) High resolution micrograph of as prepared GaON annealed at 500 °C for 4 hours, the image shows thickening and distortion of the nanoflakes.

Our observations are further supported with XRD analysis carried out for each sample as can be seen in Fig. S2. The FE-SEM micrographs are presented in Fig. S1, where Fig. S1 (a) shows the low resolution FE-SEM micrograph of GaON at reaction time of 3 hours. It is quite evident that not as much of GaON nanosheets like morphology is observed, this indicated that the reaction has just initiated and requires more time for completion. XRD analysis presented in Fig.S2 also support this observation where few low intensity peaks can be seen. Fig. S1. (b) presents the FE-SEM image of GaON prepared at 6 hours, it also shows undeveloped GaON nanosheets. Furthermore, the low intensity peaks in XRD spectra of GaON obtained from 6 hours sample as shown in Fig. S2, is also complimenting incomplete GaON nanosheets formation hence required further reaction time. The Fig. S1 (c) contains FE-SEM of GaON synthesized at reaction time of 12 hours. A better morphology is perceived but still no uniform distribution is seen under FE-SEM, in addition to this, mostly growing nanosheets are observed. The XRD spectra also showed better peak intensities as of Fig.S2. The FE-SEM image of GaON prepared at 24 hours is shown in Fig. S1 (d), the low resolution micrograph displayed GaON nanoflakes formation with uniform distribution. Their high resolution images are presented in Fig. 1 (manuscript), a clear arrangement of nanosheets with variable thickness and length are observed. The XRD analysis of GaON prepared at 24 hours also showed sharp peaks which further corroborated its formation at this reaction time and temperature. Based on these observations, we concluded to perform PEC measurements with 12 hours and 24 hours samples.

The PEC results presented in Fig. 8, showed enhanced PEC properties of as synthesized GaON nanosheets at 24 hours and 180°Cas compared to other samples. In addition to evaluate the photoelectrochemical properties of the selected material, the GaON prepared at 24 hours is further annealed at 500°C for four hours. The FE-SEM micrographs in Fig. S1 (e) & (f) show deformation of GaON nanosheets as well as thickening and distortion of the nanoflakes. The XRD analysis of annealed sample also showed low intensity peaks as compared to XRD observed for as prepared GaON at 24 hours. This is assumed to happen because of change in degree of crystallinity as well as phase at high temperature annealing. The PEC measurement of annealed sample showed diminishing response to light as presented in Fig.8 (manuscript). The most probable reason for this recital is the deformed morphology,thickening and distortion of the GaON nanosheets at high temperature.

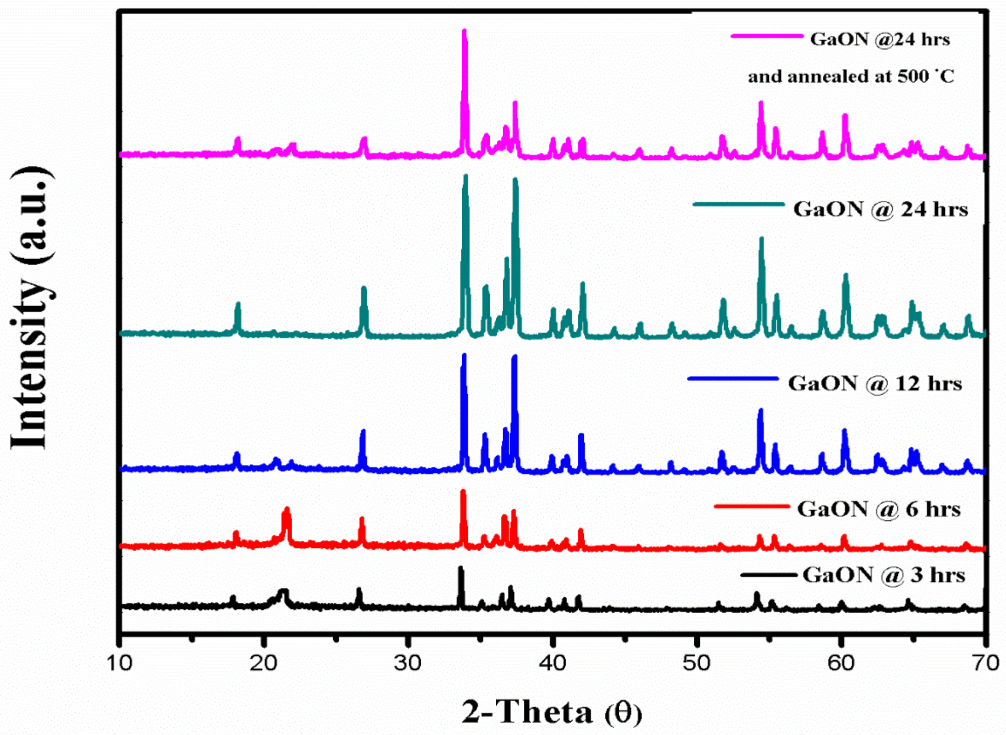

Figure S2: Comparative XRD analysis of GaON prepared at different reaction conditions.

2. XPS photoelectron spectroscopy.

Fig.S3 (a),(b) and (c) show the XPS obtained from the as prepared GaON nanosheets to investigate and confirm the elements and their chemical states.Fig. S3(a) shows the overall survey of the samples and the peaks are assigned accordingly, while Fig. S3(b) and Fig.S3(c) provide the extended information of the labelled and fitted peaks in the survey spectrum which and coherent with Hu et al studies for GaON[5]. They explained strong hybridization between the valence orbitals of Ga ($4s^2$) ($3d^{10}$) & ($4p^1$), N ($2s^2$) ($2p^3$) and O ($2s^2$) ($2p^4$) atoms. Their XPS chemical states can be observed in the lower part of the spectra ranging from binding energy values of 0-14 eVand assigned to hybrid Ga4p-N2p, Ga4s-N2p, and Ga4s-O2p chemical states[6-8]. This effect from XPS studies further augment that the presence of O2p orbital decreases the energy difference between the upper and lower valence levels in addition of enhancing p-d (O 2p- Ga 3d) repulsion significantly, resulted shifting in valence band level upright and causes reduction in GaON band gap.Fig.S3(c) presents chemical states of Ga 3d, the main peaks is recorded at binding energy19.77eV whereas a shoulder peak of low intensity is observed at 21.87 eV respectively. These peaks are shifted to somewhat higher values and that is obviously due to different synthetic strategies. However, these peaks show d-p repulsions between the valence energy levels of the reacting atoms. This is also supported from literature for Ga compounds[5,9-11].

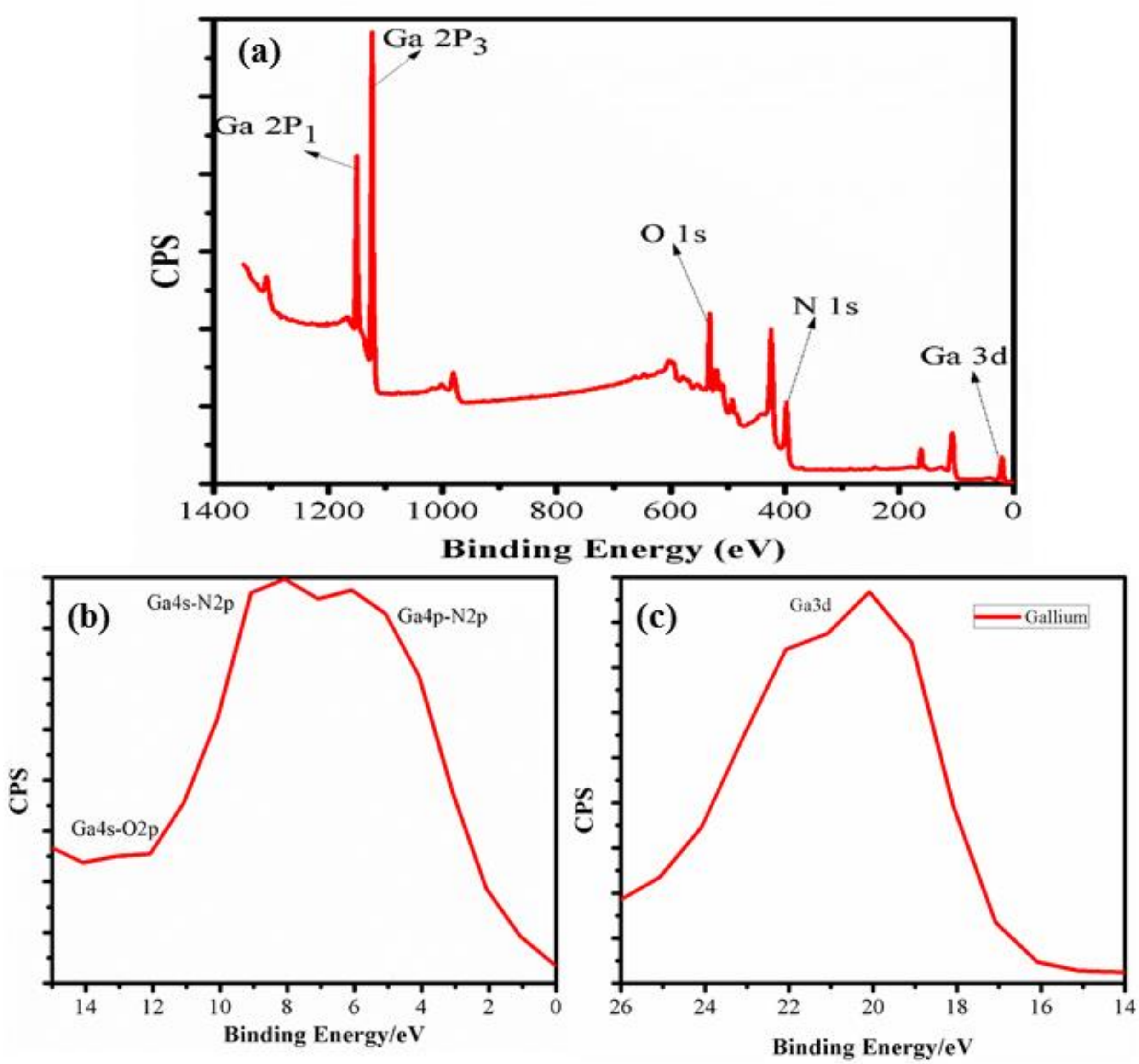

Figure S3: XPS spectrum of GaON nanosheets (a) complete survey, (b) showing valence orbitals chemical states and possible interactions (c) presenting Ga 3d state, XPS discussed in literature[5].

3. FT-IR spectroscopy.

The FT-IR spectrum of as synthesized GaON nanosheets is presented in Fig. S4. The first insight explicit the absence of OH and NH_2 functionalities that may interfere in GaON from the starting reaction precursors. The IR spectrum did not show any peaks for O-H functional group near 3500–3200 cm^{-1} as well as for N–H stretching and bending vibrational modes at (3400–3250 (m) and 1650–1580 (m) respectively. The FTIR spectrum experiment observed here show two major strong peaks at wavelength 940 cm^{-1} and 622 cm^{-1} attributed to O— Ga — N and Ga—N respectively however the onset of the selected area show the presence of two medium peaks that are assigned to Ga — O at 680 $cm^{-1}$and 655 cm^{-1}[12,13].

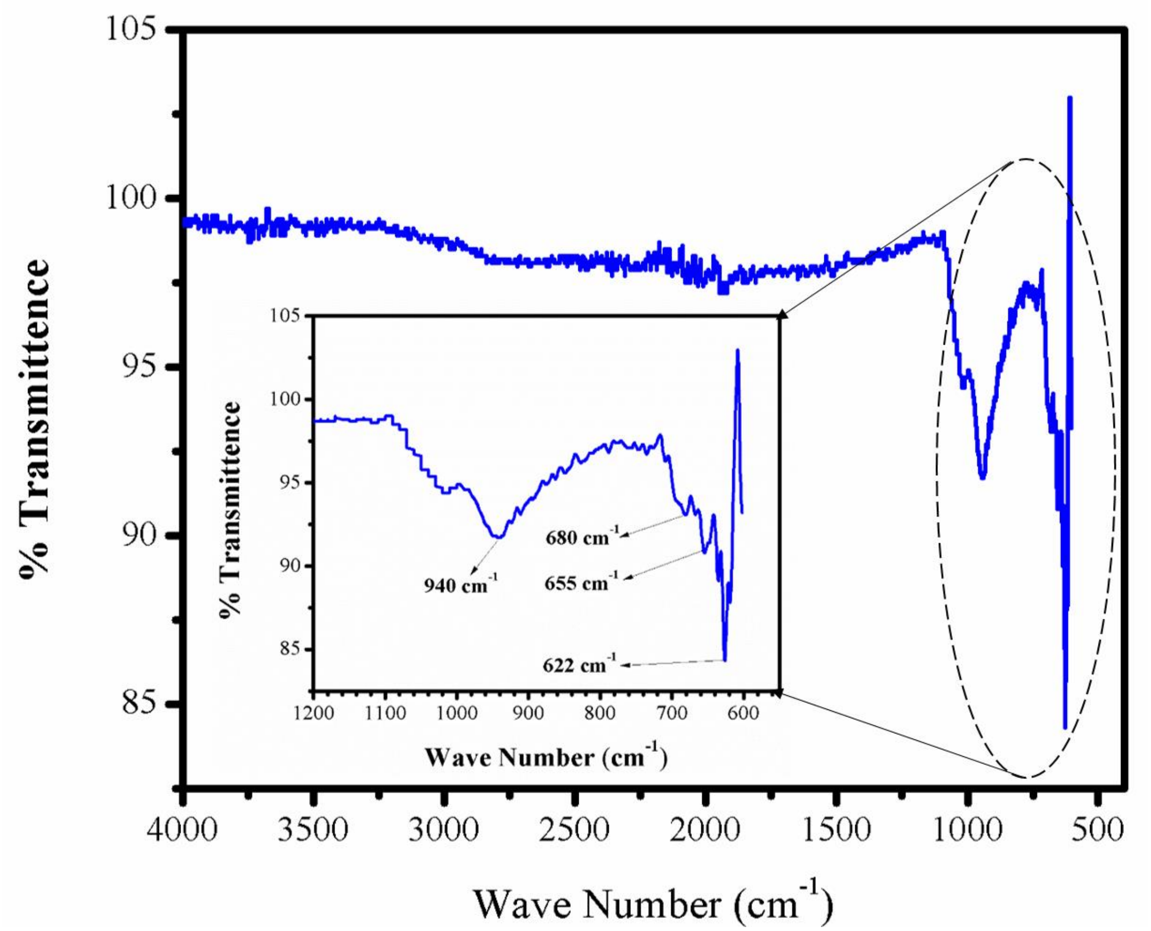

Figure S4: The FT-IR spectrum of Powder as synthesized GaON nanosheets.

4. Photoluminescence Studies of GaON Nanosheets.

The room temperature photoluminescence spectra is recorded at an excitation wavelength of 350 nm as shown in Fig. S5. The onset of the PL curve lies in the visible light region however the maxima of the PL peak is observed around ~430 nm. The onset begins at 370 nm (3.35 eV) and extended to 500nm (2.48 eV) as shown in the Fig. S5 below.The maximum excitation is observed at ~430nm (2.89 eV). Our as synthesized GaON nanosheets contains large amounts of defects and nanopores (discussed in FESEM studies) on the surface which are attributed to Ga3^{+}as well as on the anionsites in addition to disorder arrangement of oxygen and nitrogen moieties that shifted the excitation away to the band gap energy. However, the PL band appeared is due to defects related transitions between different states of disordered atoms in the material[14,15].

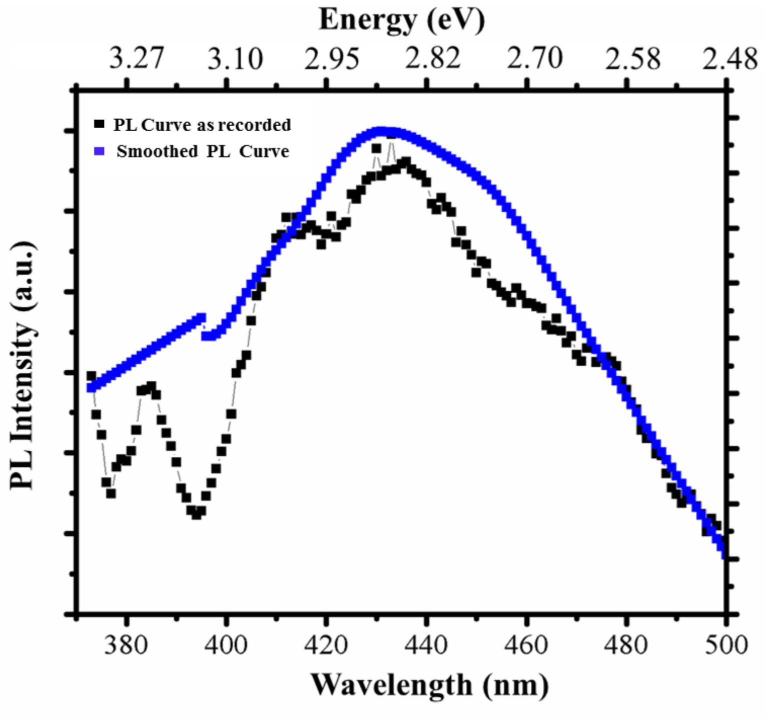

Figure S5: Photoluminescence of as prepared GaON at room temperature and excitation wavelength(350nm).

5. Stability of the GaON Nanosheets.

In chronoampereometry measurements, current density (J_p) versus time (t) were conducted to study photocurrent generation and the stability of the GaON nanosheets films coated over FTO electrodes immersed in 0.5 M Na_2SO_4. At first, it was observed that GaON photoanode showed a decaying response which gets stable upon continuous exposure to light with no significant degradation noticed within given time span as shown in Fig.7 (a)(manuscript).The first J_p spikes observed as the material was exposed to irradiation moved to higher values but later on relaxed to a stable plateau that explicit the significant stability of the GaON nanosheets with the passage of time.This kind of behavior is obvious because the GaON layer coated on FTO electrode needed some time to adjust in the solution or to establish equilibrium with the electrolyte. A selected portion of that measurement is presented here in Fig. S6 from which we can observe stability for sufficient period of time under on and off light source. The broader viewof photocurrent measurements and stability are presented in Fig. 7(b) and (c) (in manuscript) that are recorded at different applied potentials (0 V and -1.2 V). However, in allJ_p – t profiles the photocurrent peaks shifted to its normal baseline when the illumination was turned off hence showed a reversible response. The measurement showed good stability for several minutes with sufficient photocurrent generation at zero potential. Furthermore, at higher potential we observed enhanced and stable photocurrent densities in micro to miliampere range as shown in Fig. 7(c) and Fig. 8 (in manuscript) for as prepared GaON at 24 hours. This further confirms that the photocurrent produced is due to photoelectrochemical water splitting reaction. The slight upward and downward shifts or fluctuations in the photocurrent responsesmay be attributed to GaON film and electrolyte

interaction that resulted in a little depletion of GaON layer at higher potentials. It should be noted that GaON nanosheets films are simply drop casted over FTO by making a slurry of GaON in ethanol and annealed at 100°C for 2 hrs.

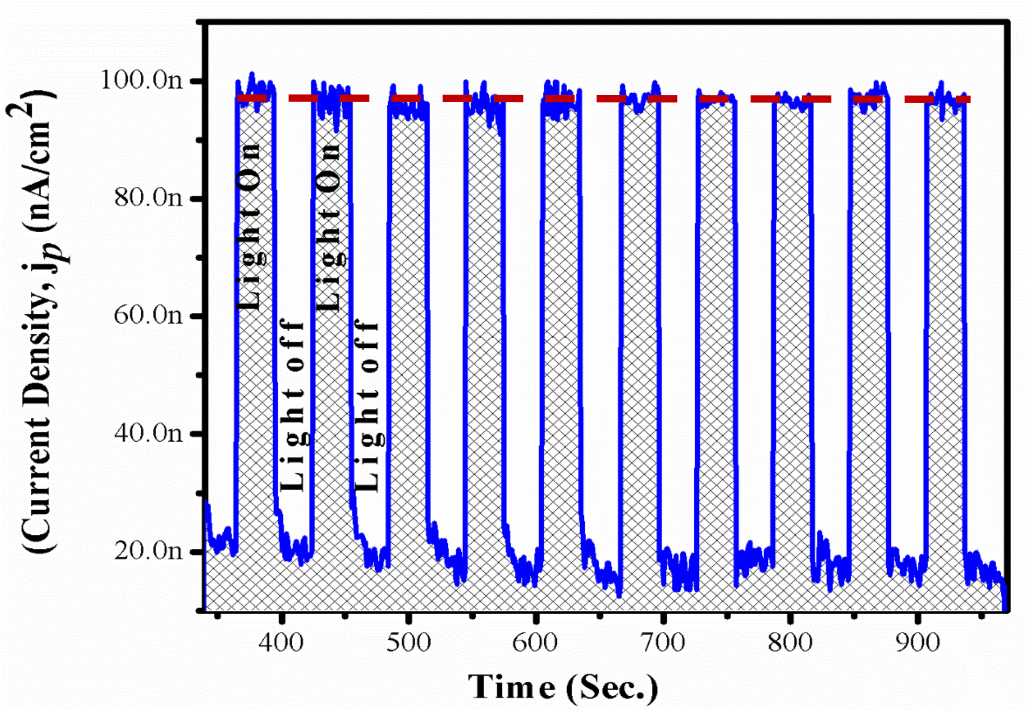

Figure S6: Photocurrent stability of GaON nanosheets observed for certain period of time.

6. Cyclic Voltammetry

Photoelectrochemical characteristics such as oxidation/reduction potentials and photocurrentetc can be evaluated by CV studies. FTO coated GaON nanosheets at 24 hours are further investigated by cyclic voltammetry in 0.5M Na_2SO_4 solution in a typical three electrode system as described in the manuscript. The potential range was selected between for -1.4 V to 0.4 V versus SCE under 1 sun simulated solar irradiation at a scan rate of 100 mV/sec. The CV

measurements are performed in chopped (off/on) mode. The FTO/ GaON nanosheets response in on/off light is presented in Fig. S7. The photocurrent generated when electrode is exposed to light is significantly higher as compared to that in dark mode. Furthermore, the reduction peaks of water due to photocatalytic activity of the as prepared material are quite evident from the cyclic voltammograms. As a result of solar water splitting, the oxidation potential is observed at -0.3 V with a photocurrent density of ± 75μA.cm^{-2}. Similarly the reduction potential is observed between -1.1 to -1.2 V and at a current density of ± 280 μAcm^{-2}.This indicated that reduction reaction i.e., hydrogen generation is more favorable by Gallium oxynitride under solar driven photocatalytic water splitting reaction.

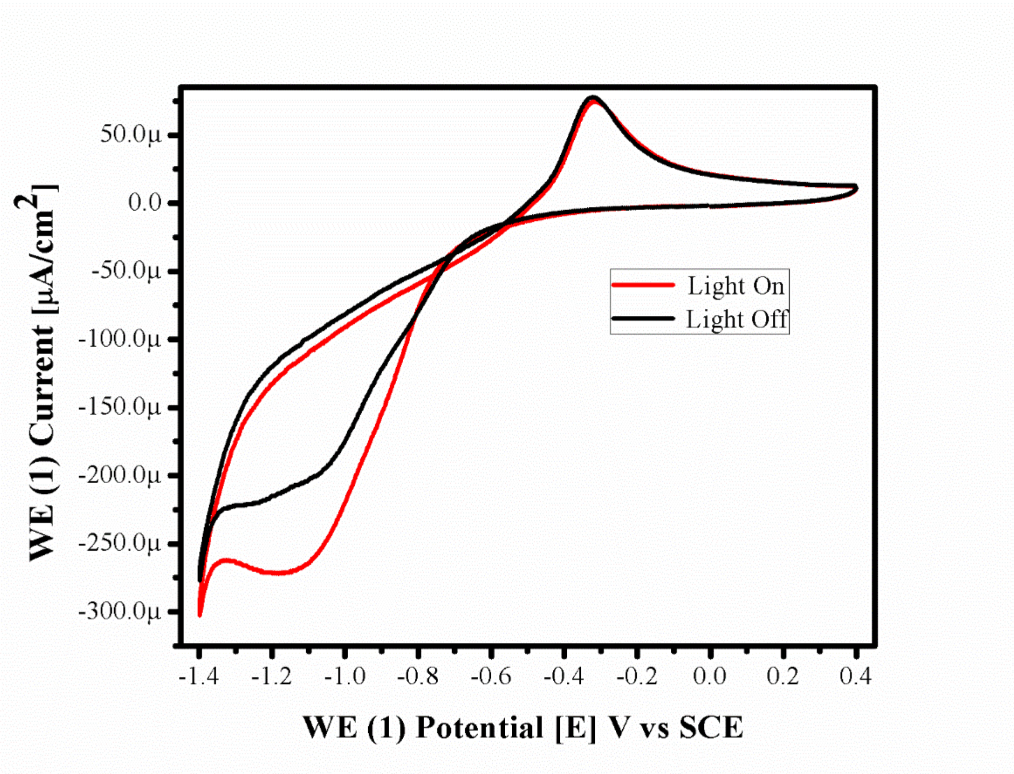

Figure S7: Cyclic voltammograms of GaON nanosheets coated FTO glass under simulated AM 1.5 solar light at the chopped (on/off) light mode with scan rate of 100mV/sec in a three electrode system, SCE, Pt-cathode and FTO/GaON as anode.

References

1 Chen, H. *et al.* In Situ XRD Studies of ZnO/GaN Mixtures at High Pressure and High Temperature: Synthesis of Zn-Rich (Ga1− x Zn x)(N1− x O x) Photocatalysts. *The Journal of Physical Chemistry C* **114**, 1809-1814 (2010).

2 Martin, M. *et al.* Thermodynamics, structure and kinetics in the system Ga–O–N. *Progress in Solid State Chemistry* **37**, 132-152, doi:http://dx.doi.org/10.1016/j.progsolidstchem.2009.11.005 (2009).

3 Kroll, P. Spinel-type gallium oxynitrides attainable at high pressure and high temperature. *Physical Review B* **72**, 144407 (2005).

4 Kinski, I. *et al.* High-pressure synthesis of a gallium oxonitride with a spinel-type structure. *Zeitschrift fur Naturforschung - Section B Journal of Chemical Sciences* **60**, 831-836 (2005).

5 Hu, C.-C. & Teng, H. Gallium Oxynitride Photocatalysts Synthesized from Ga(OH)3 for Water Splitting under Visible Light Irradiation. *The Journal of Physical Chemistry C* **114**, 20100-20106, doi:10.1021/jp1070083 (2010).

6 Martin, G. *et al.* Valence-band discontinuity between GaN and AIN measured by x-ray photoemission spectroscopy. *Journal of electronic materials* **24**, 225-227 (1995).

7 Maruyama, T. *et al.* Electronic structure of wurtzite-and zinc blende-GaN studied by angle-resolved photoemission. *Journal of electronic materials* **27**, 200-205 (1998).

8 Fiorentini, V., Methfessel, M. & Scheffler, M. Electronic and structural properties of GaN by the full-potential linear muffin-tin orbitals method: The role of the d electrons. *Physical Review B* **47**, 13353 (1993).

9 Carin, R., Deville, J. P. & Werckmann, J. An XPS study of GaN thin films on GaAs. *Surface and Interface Analysis* **16**, 65-69, doi:10.1002/sia.740160116 (1990).

10 Bhaviripudi, S., Qi, J., Hu, E. L. & Belcher, A. M. Synthesis, Characterization, and Optical Properties of Ordered Arrays of III-Nitride Nanocrystals. *Nano Letters* **7**, 3512-3517, doi:10.1021/nl072129d (2007).

11 Procop, M. XPS data for sputter-cleaned In0. 53Ga0. 47As, GaAs, and InAs surfaces. *Journal of electron spectroscopy and related phenomena* **59**, R1-R10 (1992).

12 Ganesh, V., Suresh, S., Balaji, M. & Baskar, K. Synthesis and characterization of nanocrystalline gallium nitride by nitridation of Ga-EDTA complex. *Journal of Alloys and Compounds* **498**, 52-56, doi:http://dx.doi.org/10.1016/j.jallcom.2010.03.068 (2010).

13 Qin, L., Xue, C., Duan, Y. & Shi, L. Synthesis and Characterization of Glomerate GaN Nanowires. *Nanoscale Research Letters* **4**, 584-587, doi:10.1007/s11671-009-9285-y (2009).

14 Soignard, E. *et al.* Spinel-Structured Gallium Oxynitride (Ga3O3N) Synthesis and Characterization: An Experimental and Theoretical Study. *Chemistry of Materials* **17**, 5465-5472, doi:10.1021/cm051224p (2005).

15 Oberländer, A., Kinski, I., Zhu, W., Pezzotti, G. & Michaelis, A. Structure and optical properties of cubic gallium oxynitride synthesized by solvothermal route. *Journal of Solid State Chemistry* **200**, 221-226, doi:http://dx.doi.org/10.1016/j.jssc.2013.01.040 (2013).
